# Choroidal thickness and choriocapillaris vascular density in myopic anisometropia

**DOI:** 10.1186/s40662-021-00269-9

**Published:** 2021-12-02

**Authors:** Xinting Liu, Zhiyi Lin, Feifu Wang, Xiaoyi Peng, Wenwen He, Dan Chen, Meixiao Shen, Fan Lu, Jun Jiang

**Affiliations:** School of Ophthalmology and Optometry, Eye Hospital, Wenzhou Medical University, 270 Xueyuan Road, Wenzhou, 325027 Zhejiang China

**Keywords:** Myopic anisometropia, Choroidal choriocapillaris, Choroidal thickness

## Abstract

**Background:**

This study aims to examine interocular differences in the choroidal thickness and vascular density of the choriocapillaris in anisometropic myopes and to further explore the relationship between choroidal blood flow and myopia.

**Methods:**

The sample comprised 44 participants with anisometropic myopia, aged 9 to 18 years, with normal best-corrected visual acuity. All participants underwent a series of examinations, including spherical equivalent refraction (SER) and axial length (AL), measured by a Lenstar optical biometer and optical coherence tomography angiography (OCTA) scanner. OCT measured the choroidal thickness, vascular density, and flow voids of the choriocapillaris, and a customized algorithm was implemented in MATLAB R2017a with the post-correction of AL. The choroidal thickness was measured at the fovea and 0.5, 1.0, 1.5, 2.0, 2.5, and 3.0 mm nasally, temporally, inferiorly, and superiorly to the fovea. The vascular density and the flow voids of the choriocapillaris were measured at a 0.6-mm-diameter central circle, and the 0.6–2.5 mm diameter circle in the nasal, temporal, inferior, and superior regions. Repeated-measured ANOVAs were used to analyze the interocular differences. Partial correlations with the K value and age adjustments were used to study the relationships between the choroidal thickness, the choriocapillaris vascular density and flow voids, the SER and AL.

**Results:**

The choroidal thickness of the more myopic eyes was significantly thinner than less myopic eyes (*P* ≤ 0.001), and the flow voids in the more myopic eyes were more than less myopic eyes (*P* = 0.002). There was no significant difference in the vascular density of the choriocapillaris between the more and less myopic eyes (*P* = 0.525). However, when anisometropia was more than 1.50 D, the vascular density of choriocapillaris in the more myopic eyes was significantly less than the less myopic eyes (*P* = 0.026). The interocular difference of the choroidal thickness was significantly correlated with the interocular difference in SER and AL in the center, superior, and inferior regions but not in the nasal or temporal regions. The interocular differences of the vascular density and the flow voids of the choriocapillaris were not correlated with the interocular difference of SER and AL.

**Conclusions:**

The choroidal thickness is thinner in the more myopic eyes. The flow void is increased, and the vascular density of the choriocapillaris is reduced in the more myopic eyes of children with anisometropia exceeding 1.50 D.

## Introduction

In recent years, the prevalence of myopia among children and adolescents has continued to increase substantially. Myopia has a significant impact on ocular health and quality of life, and it is estimated that the number of people with myopia will reach about 4 billion by 2050 [1–3]. However, the exact mechanisms underlying the onset and progression of myopia remain unclear. Several studies have found that the choroid plays an important role in the development of myopia, as evidenced by the bidirectional changes in choroidal thickness in response to imposed myopic or hyperopic defocusing, termed “choroidal accommodation” [4–8]. After wearing orthokeratology lenses for a period as short as 3 to 4 weeks, myopic patients exhibited choroidal thickening. Further, after close-range reading for a short period, choroidal thickness is decreased [9–11]. This indicates that choroidal mechanisms are involved in regulating the refractive error in the human eye. The choroid is made of highly vascularized tissue; its primary function is to act as a source of nutrients and oxygen for the outer retina and sclera. Recently, several studies have shown that the thinning of the choroid and reduced perfusion occur early in myopia development, leading to an insufficient supply of oxygen and nutrients to the nearby sclera and ultimately causing axial elongation and myopia [12–14]. Therefore, it is imperative to further investigate the effect of changes in choroidal blood flow and its association with myopia in humans to understand the role of choroidal blood flow in the pathogenesis of myopia.

Several clinical studies have evaluated blood perfusion of the choriocapillaris in patients with high myopia and other retinal diseases using optical coherence tomography angiography (OCTA) [15–19]. However, the results of these studies were inconsistent. While some studies reported an increase in the total and average area of flow voids in the choriocapillaris [20, 21], others did not find any significant differences in the choriocapillaris perfusion area between myopic and non-myopic eyes [22]. One reason for this inconsistency might be the significant variability in individual choroidal structure, making it challenging to analyze the correlations between the choroidal vascular density and both the development of the refractive error and axial length (AL). Individuals with anisometropia, who have significantly different refractive powers in each eye, are a unique and powerful study population that can be used to address this problem. This is because comparing the eyes of the same individual enables us to control the effects of age, gender, and genetic and environmental factors. Vincent et al. reported that the interocular differences in refraction in anisometropia are mainly due to differences in the posterior segment of the eye, such as the vitreous chamber depth (VCD), AL, and choroidal thickness. Patients exhibit similar profiles on other ocular biometric parameters, such as the corneal curvature, anterior chamber depth (ACD), and crystalline lens power, and thus minimized the risks of confounding variables [23]. One recent study on anisometropic patients only examined choroidal thickness and did not investigate choriocapillaris changes [24]. Although other studies have investigated the choriocapillaris in adult and pediatric patients with myopic anisometropia, these studies focused on the choriocapillaris flow voids and other parameters but did not examine choriocapillaris vascular density changes [25, 26]. Therefore, our study examined interocular differences in choroidal thickness, vascular density, and flow voids of the choriocapillaris in anisometropic myopes to further explore the relationship between choroidal blood flow and myopia in young patients.

## Methods

### Participants

This cross-sectional study included 44 anisometropic myopes aged 9 to 18 years (14.09 ± 2.35 years) recruited from the Eye Hospital of Wenzhou Medical University. All subjects had interocular differences in their spherical equivalent refraction (SER) of at least 1.00 D, with a best-corrected logMAR visual acuity of 0 (20/20) or better in each eye. For all patients, the SER of the more myopic eye was less than − 8.00 D, the SER of the less myopic eye was less than − 6.00 D, and astigmatism was less than 2.00 D. The participants underwent a series of screening examinations, including subjective refraction, binocular vision, intraocular pressure (IOP), and ocular health status to rule out contraindications and ensure their eligibility for the study. All participants were free of ocular and systemic diseases, had no history of ocular surgery or trauma, had never worn orthokeratology lenses or multifocal soft contact lenses, and had never used low-concentration atropine. This study was approved by the Office of Research Ethics Committee at the Eye Hospital of Wenzhou Medical University and was performed in line with the tenets of the Helsinki Declaration. Written informed consent was obtained from all participants and their legal guardians.

### Screening and imaging

Each subject’s refractive error was measured without cycloplegia. After auto-refraction, subjective refraction was performed. All subjects underwent an examination by an experienced ophthalmologist at the Eye Hospital of Wenzhou Medical University. SER was measured as the spherical power plus half of the cylindrical power.

The AL (defined as the distance from the front of the central cornea to the front of the retinal pigment epithelium), keratometry (K), central corneal thickness (CCT), ACD, and lens thickness (LT) were measured using a Lenstar LS 900 optical biometer (Haag-Streit, Streit, Koeniz, Switzerland). The VCD was calculated using the following formula: VCD = AL − CCT − ACD − LT. Each eye was measured at least five times until the difference (maximum value − minimum value) in AL over five consecutive measurements were within 20 μm.

Choroidal structure images were acquired using a spectral domain optical coherence tomography (SD-OCT) scanner (Optovue RTvue OCT instrument). The light wavelength was 840 ± 10 nm, and the scanning speed was 70,000 A-scan/s, providing an axial resolution of 5 μm and a lateral resolution of 15 μm. The horizontal and vertical choroidal thickness images were scanned centering on the fovea using the Enhance HD line mode. A total of 250 frames averaged for each B-scan. The scan line length was 9 mm, the “chorioretinal” mode was selected, and the images were collected and recorded when the signal strength was greater than 40.

Images of the choriocapillaris were obtained in the Angio Retina (3 × 3 mm) mode, centered on the fovea. This refers to each B-scan containing 304 A-scans of various meridians in this 3 × 3 mm square area. The scans were repeated 24 times in two directions and were automatically integrated when the scanning quality was no worse than 6.

We did not exclude participants with habitual uncorrected, undercorrected, or overcorrected refractive errors. However, we completed the refraction before performing SD-OCT and OCTA. The subjects were then directed to have a rest and watched a 5-m distanced video on television for 10 min with full-distance sphere and astigmatism correction. This was done to eliminate the effect of any previous visual stimuli on the choroid, including high accommodation [9] and sphere and astigmatism defocus [5, 27]. All measurements were conducted between 9:30 and 11:30 a.m. in a dark room to decrease the influence of diurnal variation on the choroid [28].

### Image data processing

The choroidal thickness and vascular density of the choriocapillaris were extracted and measured by a customized algorithm using MATLAB R2017a, with the post-correction of AL. Segmentations of retinal pigment epithelial (RPE)-Bruch’s membrane complex and the choroid-sclera interfaces were adjusted manually by a trained examiner.

The ocular magnification was adjusted using Bennett’s formula, and all images were corrected using the AL. The relationship between the OCT image measurements and the actual scan diameter was expressed by the formula *t* = *p* × *q* × *s*, where *t* represents the actual scan diameter, *p* represents the magnification factor determined by the camera of the OCT imaging system, *q* represents the magnification factor concerning the eye, and *s* represents the original measurement value obtained from the OCT image. The *q* was determined according to the equation *q* = 0.01306 × (AL − 1.82) [29].

**Fig. 1:**
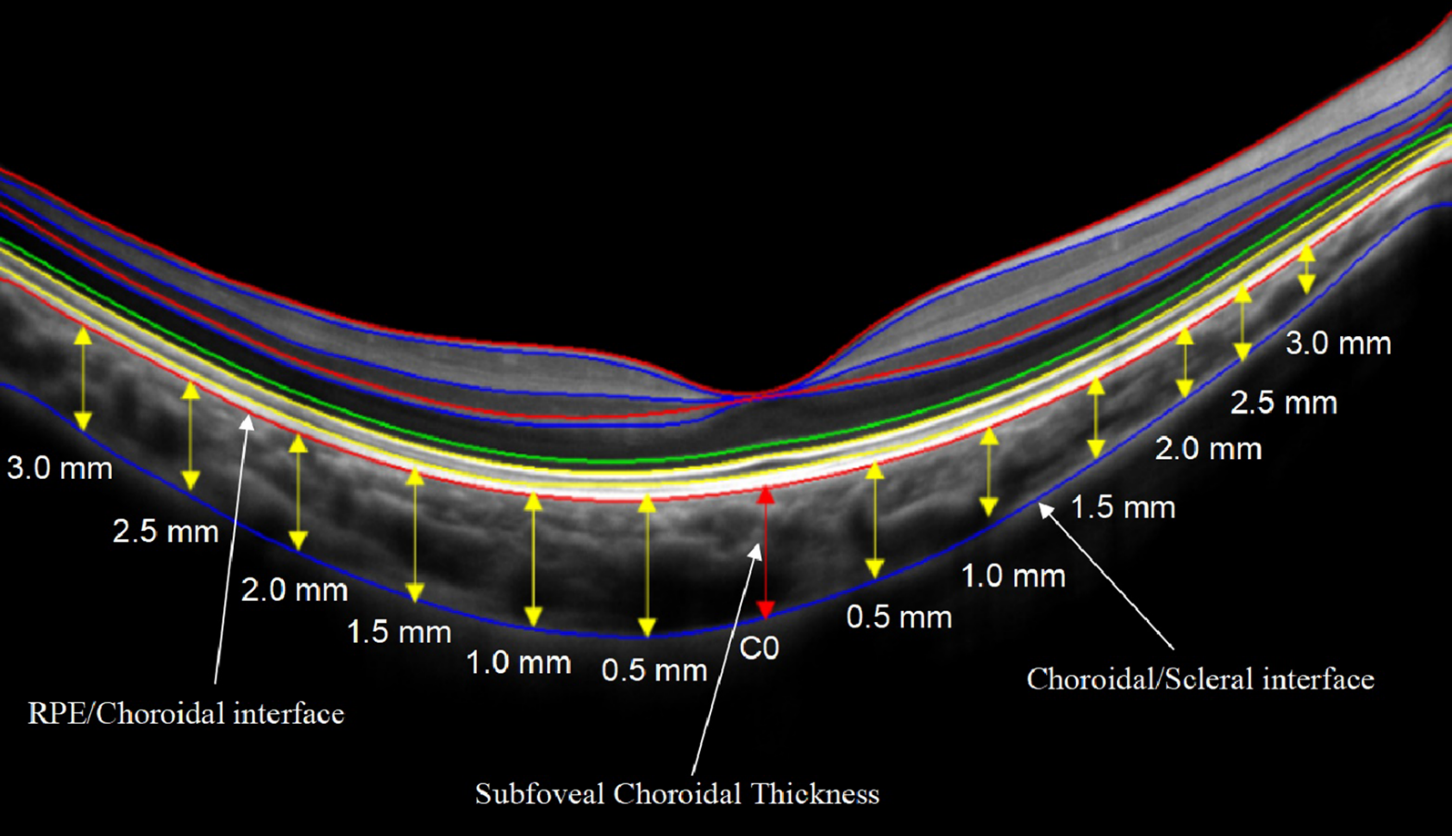
Choroidal thickness measurements on Optovue RTvue OCT enhance-line scans

Choroidal thickness was defined as the axial distance from the outside of the retina pigment epithelium to the choroid sclera boundary. The measurement position was at the fovea, and it was 0.5, 1.0, 1.5, 2.0, 2.5, and 3.0 mm nasally, temporally, inferiorly, and superiorly to the fovea (Fig. 1) [30]. The choroid thicknesses were analyzed in five regions that corresponded with the 3.0-mm-diameter region of the vascular density and flow voids of the choriocapillaris. The subfoveal choroidal thickness (SFCT) was taken from the average value of the SFCT on the horizontal and vertical meridians and the nasal, temporal, superior, and inferior regions (the mean value of the 1.5-, 1.0-, and 0.5-mm points in each region).

**Fig. 2:**
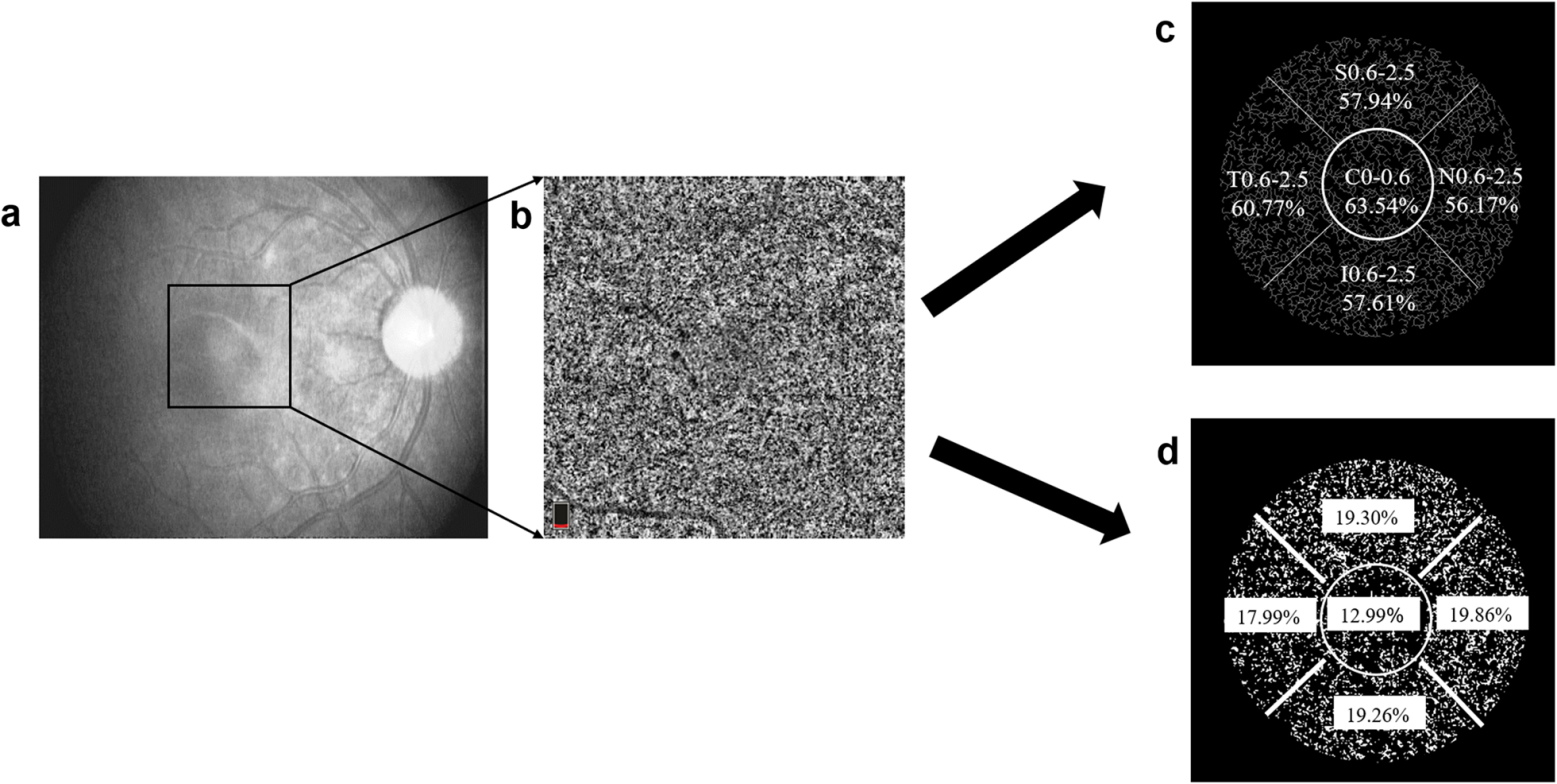
Vascular density and flow void of the choriocapillaris measurements on Optovue RTvue OCT, Angio Retina mode (3 × 3 mm). **a** En face OCTA image. **b** OCTA choriocapillaris image. **c** Vascular density (white) isolated from the total area. **d** The flow voids (white) isolated from the total area. C0.6, the central foveal area (0–0.6 mm); N0.6–2.5, T0.6–2.5, I0.6–2.5, and S0.6–2.5 represent the 0.6–2.5 mm region in nasal, temporal, inferior, and superior, respectively. The values of the choriocapillaris vascular density and the flow voids of one subject in different sections are shown in **c** and **d**

The choriocapillaris was defined as the structure located from 9 μm above (upper border) to 31 μm below (lower border) Bruch’s membrane and was automatically recognized by the OCT system. A low brightness threshold (average pixel brightness of the superficial layer of the retina in the 1–3 mm region + 1 SD) was used to eliminate artifacts caused by larger blood vessels in the retina. Further, the structures above this brightness threshold were defined as vascular tissue. The density of the choriocapillaris was calculated as the ratio of the pixel areas of the choriocapillaris vessels divided by the total area of the regions. The choriocapillaris vessels were selected using the threshold function of the software. The choriocapillaris flow void was defined as a percentage between the region absent from the flow and the total scanned region. A thresholding method was used to calculate the percentage of flow voids, previously described by Zhang et al. [31]. The thresholding methods used for the density of the choriocapillaris and flow void were different, so these parameters did not complement each other perfectly. The image was cropped using a multiple-step approach, involving a 0.6-mm-diameter central circle and a 2.5-mm-diameter circle (without the inner central 0.6 mm circle). Both circles are centered on the foveola marked on the image. The 0.6–2.5 mm circle is divided into the nasal, temporal, inferior, and superior areas (Fig. 2) [15].

The choroidal thickness, the vascular density, and flow void of the choriocapillaris were measured by two independent observers, and the average of the measurements from the two observers was used for the analysis.

### Statistical analysis

Statistical analysis was performed using SPSS software (version 22.0; IBM, Armonk, NY, USA). Descriptive statistics were determined as means ± standard deviations. A Shapiro–Wilk test was used for data normality analysis. Paired t-tests were used to analyze the interocular differences in SER, the biometric data collected with the LS900, and the entire vascular area of the choriocapillaris. Independent sample t-tests were used to analyze differences between the two different anisometropia groups in age, SER, and the biometric data collected with the LS900. Further, a chi-square test was used to compare gender ratios among different groups. Three-way repeated measures analysis of variances (ANOVA) was performed to compare the choroidal thickness between the fellow eyes with eyes (2 levels), points (13 levels), and meridian (2 levels) as within-subject factors. A two-way repeated measures ANOVA was conducted to test choroidal thickness, vascular density, and flow voids of the choriocapillaris between the fellow eyes (2 levels) and regions (5 levels) as within-subject factors. Bonferroni tests were applied to all multiple pairwise comparisons. Partial correlations with the K value and age adjustments were used to study the relationships among choroidal thickness, vascular density, and flow voids of the choriocapillaris, SER, and AL. Bonferroni adjustments were also used in multiple correlation analyses at different regions. *P* < 0.05 indicated a statistically significant difference.

## Results

### General characteristics

Of the 44 participants included in the analyses, there were 19 males and 25 females, with a mean age of 14.09 ± 2.35 years. There were no significant differences between males and females with regards to age and the optical parameters (AL, SER, VDC, and K mean of the interocular difference). As expected, among the 44 subjects, AL and VCD of the more myopic eyes were significantly longer than for less myopic eyes (all *P* < 0.05; Table 1).

**Table 1:** Characteristics of anisometropic subjects

Parameter**s**	More myopic eye	Less myopic eye	*P*
SER (D)	− 3.18 ± 1.44 (− 7.5 to − 1.25)	− 1.47 ± 1.40 (− 5.25 to 0)	< 0.001
AL (mm)	25.23 ± 1.03 (23.45 to 28.38)	24.49 ± 0.91 (22.94 to 27.49)	< 0.001
VCD (mm)	18.13 ± 1.09 (16.47 to 21.37)	17.35 ± 0.90 (15.98 to 20.48)	< 0.001
K mean (D)	42.89 ± 1.37 (39.03 to 45.94)	42.80 ± 1.39 (38.92 to 45.5)	0.063

*SER* spherical equivalent refraction; *D* diopter; *AL* axial length; *VCD* vitreous chamber depth; *K* keratometry

### Repeatability of choroidal thickness and vascular density measurements

The image processing results obtained by the two independent observers concerning choroidal thickness and vascular density were closely related. In all measurement areas, the intraclass correlation coefficient (ICC) of the choroidal thickness ranged from 0.92 to 0.97, the vascular density of the choriocapillaris ranged from 0.93 to 0.98, and the flow voids ranged from 0.98 to 0.99. The coefficient of repeatability (COR) of the choroidal thickness ranged from 22.74 to 38.02 μm, the vascular density of the choriocapillaris were from 4.78 to 5.19%, and the flow voids were from 0.20 to 0.74% (Table 2).

**Table 2:** The repeatability of choroidal thickness, vascular density, and flow voids of the choriocapillaris measurements

Parameters	Average measurement of examiner 1 (mean ± SD)	Average measurement of examiner 2 (mean ± SD)	ICC	COR
Choroidal thickness (μm)				
Center	235.59 ± 45.22	228.09 ± 48.02	0.97	23.52
Superior	245.59 ± 45.35	243.54 ± 48.27	0.93	34.50
Inferior	240.90 ± 47.32	235.48 ± 48.58	0.95	30.97
Nasal	203.28 ± 42.73	201.04 ± 46.80	0.97	22.74
Temporal	241.88 ± 46.75	243.65 ± 49.81	0.92	38.02
Vascular density (%)				
C0–0.6	72.41 ± 10.90	72.52 ± 10.93	0.98	4.78
S0.6–2.5	63.20 ± 7.83	63.29 ± 7.14	0.94	5.12
I0.6–2.5	64.30 ± 7.25	64.38 ± 6.50	0.93	5.19
N0.6–2.5	65.28 ± 7.29	65.42 ± 6.45	0.93	5.08
T0.6–2.5	64.41 ± 7.36	64.41 ± 6.65	0.93	5.17
Flow void (%)				
C0–0.6	10.72 ± 2.10	10.67 ± 1.90	0.98	0.74
S0.6–2.5	20.92 ± 1.75	20.84 ± 1.78	0.99	0.51
I0.6–2.5	19.74 ± 0.74	19.77 ± 0.69	0.99	0.20
N0.6–2.5	19.02 ± 1.54	19.04 ± 1.60	0.98	0.55
T0.6–2.5	20.17 ± 1.90	20.29 ± 2.00	0.98	0.71

C0–0.6, represents the central foveal area (0–0.6 mm). N0.6–2.5, T0.6–2.5, I0.6–2.5, and S0.6–2.5 represent the 0.6–2.5 mm region in nasal, temporal, inferior, and superior, respectively

*ICC* intraclass correlation coefficient; *COR* coefficient of repeatability

### Interocular differences of choroidal thickness and vascular density in anisometropic patients

**Fig. 3:**
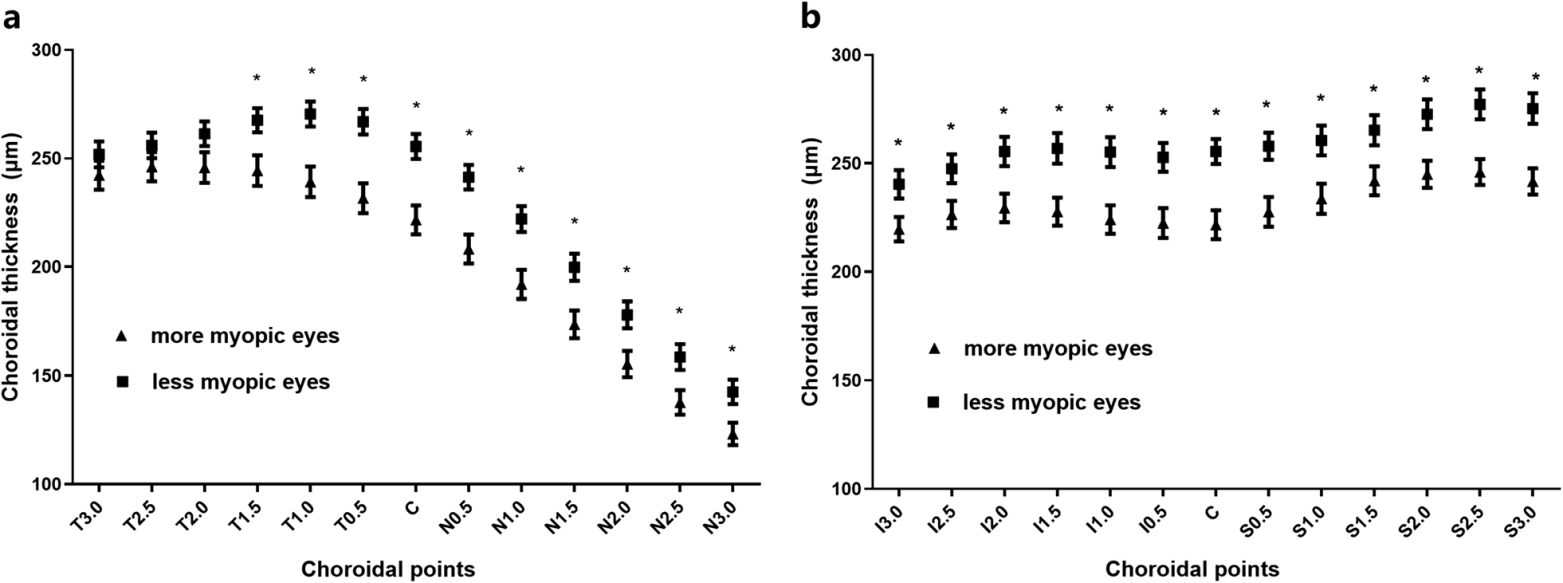
Mean choroidal thickness of the more and less myopic eyes of all participants. **a** Choroidal thickness in horizontal B-scan at T3.0, T2.5, T2.0, T1.5, T1.0, T0.5, C, N0.5, N1.0, N1.5, N2.0, N2.5 and N3.0; **b** Choroidal thickness in vertical B-scan at I3.0, I2.5, I2.0, I1.5, I1.0, I0.5, C, S0.5, S1.0, S1.5, S2.0, S2.5 and S3.0. Data are expressed as the mean ± SEM. * Represents a statistically significant difference

The choroidal thickness in the horizontal of the more myopic eyes was significantly thinner than that of less myopic eyes at T1.5, T1.0, T0.5, C, N0.5, N1.0, N1.5, N2.0, N2.5 and N3.0 (all *P* ≤ 0.001, Fig. 3a). In the vertical, the choroidal thickness was significantly thinner in the more myopic eyes at all the points (all P ≤ 0.001, Fig. 3b). The interocular differences of choroidal thicknesses ranged from 11.50 to 36.86 μm.

**Fig. 4:**
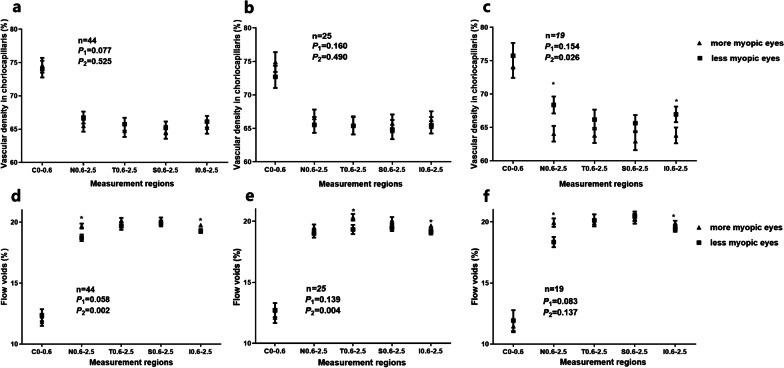
Vascular density and flow void of the choriocapillaris for the more and less myopic eyes of anisometropic patients. **a** Vascular density for all subjects in different areas. **b** Vascular density for subjects with an anisometropia value of less than 1.50 D. **c** Vascular density for subjects with an anisometropia value of more than 1.50 D. **d** Flow void for all subjects in different areas. **e** Flow void for subjects with an anisometropia value of less than 1.50 D. **f** Flow void for subjects with an anisometropia value of more than 1.50 D. Data are expressed as the mean ± SEM. * Represents a statistically significant difference. *P1*, the *P* value of the eye and region interaction; *P2*, the *P* value of the main effect of eye

However, there was no significant difference in the vascular density of the choriocapillaris (averaged across the whole 2.5 mm region) between the more and less myopic eyes of the same patient (*P* = 0.525, Fig. 4a). Further, there was no significant interaction between eyes and regions (*P* = 0.077). Further analysis showed that when anisometropia was greater than 1.50 D, the vascular density of the choriocapillaris in the more myopic eyes was significantly less than the less myopic eyes (more myopia − less myopia: − 2.97 ± 4.96%, the mean difference averaged across the whole regions, *P* = 0.026, Fig. 4c). Additionally, there was no interaction between eyes and regions (*P* = 0.154).

The flow voids of the more myopic eyes were significantly larger than less myopic eyes (*P* = 0.002, Fig. 4d), and there was no interaction between eyes and regions (*P* = 0.058). The interocular difference ranged from 12.37 to 19.90%.

In Table 3, the interocular differences of the choroidal thickness of the anisometropia > 1.50 D were more than the anisometropia < 1.50 D in all the regions, except the temporal area. The vascular density had significant differences between the anisometropia groups cutoff from 1.50 D in the N0.6–2.5 mm and I0.6–2.5 mm areas.

**Table 3:** The characteristics and interocular differences of choroidal thickness, vascular density, and flow void in anisometropia cutoff from 1.50 D

Parameters	Anisometropia more than 1.50 D	Anisometropia less than 1.50 D	*P* value
Number	19	25	
Age (years)	14.84 ± 2.22	13.52 ± 2.33	0.064
M:F	10:9	9:16	0.270
SER (D)	2.34 ± 0.62	1.26 ± 0.23	< 0.001
AL (mm)	0.94 ± 0.38	0.58 ± 0.38	< 0.001
VCD (mm)	1.08 ± 0.70	0.56 ± 0.20	0.001
K mean (D)	0.21 ± 0.37	0.12 ± 0.28	0.347
Choroidal thickness (μm)			
Center	− 54.98 ± 44.97	− 17.86 ± 29.23	0.002
Nasal	− 46.04 ± 45.44	− 17.47 ± 35.66	0.024
Temporal	− 38.38 ± 41.81	− 23.46 ± 32.67	0.191
Superior	− 49.30 ± 43.76	− 9.80 ± 32.66	0.001
Inferior	− 51.91 ± 42.06	− 13.70 ± 27.41	0.001
Vascular density (%)			
C0–0.6	− 1.60 ± 6.04	2.22 ± 8.46	0.103
N0.6–2.5	− 4.31 ± 5.83	1.07 ± 6.70	0.008
T0.6–2.5	− 2.38 ± 6.41	0.05 ± 8.21	0.293
S0.6–2.5	− 2.70 ± 5.65	0.93 ± 7.79	0.094
I0.6–2.5	− 3.14 ± 5.10	0.82 ± 7.25	0.049
Flow void (%)			
C0–0.6	− 0.46 ± 2.67	− 0.60 ± 2.87	0.868
N0.6–2.5	− 1.61 ± 2.07	0.48 ± 1.77	0.059
T0.6–2.5	0.21 ± 2.31	0.99 ± 2.01	0.072
S0.6–2.5	− 0.18 ± 2.19	0.59 ± 1.53	0.174
I0.6–2.5	0.49 ± 0.98	0.51 ± 1.07	0.951

C0–0.6, represents the central foveal area (0–0.6 mm). N0.6–2.5, T0.6–2.5, I0.6–2.5, and S0.6–2.5 represent the 0.6–2.5 mm region in nasal, temporal, inferior, and superior, respectively

*M* male; *F* female; *SER* spherical equivalent refraction; *D* diopter; *AL* axial length; *VCD* vitreous chamber depth; *K* keratometry

### Relationships between interocular differences in the choroid thickness, vascular density, flow void, SER, and AL

Factors related to the interocular differences in the choroid thickness, vascular density, and flow void of the choriocapillaris were also analyzed. The interocular difference of choroidal thickness was significantly correlated with the interocular difference in SER and AL in the center, superior, and inferior areas but not the nasal or temporal areas. The interocular differences of vascular density and flow voids of the choriocapillaris were not correlated with the interocular difference of SER and AL. Moreover, the interocular difference of the flow voids in the superior area was significantly correlated with VCD (Table 4).

**Table 4:** The relationship between interocular difference of choroid thickness, vascular density, and flow voids of the choriocapillaris with interocular difference of SER, AL and VCD

Parameters	SER		AL		VCD	
	r	*P*	r	*P*	r	*P*
Choroidal thickness						
Center	0.481	0.001*	− 0.521	< 0.001*	− 0.350	0.023
Nasal	0.364	0.018	− 0.387	0.011	− 0.237	0.305
Temporal	0.235	0.133	− 0.362	0.019	− 0.237	0.130
Superior	0.407	0.008*	− 0.446	0.003*	− 0.434	0.004*
Inferior	0.560	< 0.001*	− 0.592	< 0.001*	− 0.534	< 0.001*
Average	0.458	< 0.001^#^	− 0.515	< 0.001^#^	− 4.000	0.009^#^
Vascular density						
C0–0.6	0.144	0.363	0.016	0.920	− 0.049	0.760
N0.6–2.5	0.372	0.015	− 0.221	0.160	0.296	0.057
T0.6–2.5	0.149	0.347	− 0.098	0.538	− 0.257	0.100
S0.6–2.5	0.211	0.179	− 0.048	0.763	− 0.152	0.337
I0.6–2.5	0.281	0.071	− 0.156	0.324	− 0.244	0.119
Average	0.244	0.120	− 0.105	0.509	− 0.211	0.180
Flow voids						
C0–0.6	0.035	0.826	− 0.065	0.682	− 0.326	0.035
N0.6–2.5	− 0.271	0.083	0.212	0.178	0.367	0.017
T0.6–2.5	0.253	0.106	− 0.036	0.823	0.028	0.861
S0.6–2.5	0.177	0.263	− 0.183	0.245	− 0.410	0.007*
I0.6–2.5	− 0.022	0.889	− 0.109	0.491	0.171	0.278
Average	0.143	0.367	− 0.032	0.841	− 0.247	0.114

C0–0.6 represents the central foveal area (0–0.6 mm). N0.6–2.5, T0.6–2.5, I0.6–2.5, and S0.6–2.5 represent the 0.6–2.5 mm region in nasal, temporal, inferior, and superior, respectively

*SER* spherical equivalent refraction; *D* diopter; *AL* axial length; *VCD* vitreous chamber depth

*After application of the Bonferroni adjustment for multiple correlation analyses, correlations were significant with a *P* < 0.01

^#^Correlations were significant with a *P* < 0.05

## Discussion

This study examined interocular differences in the choroidal thickness, vascular density, and flow voids of the choriocapillaris in anisometropic participants. The results revealed that the more myopic eyes had a thinner choroidal thickness compared to the less myopic eyes. However, there was no significant difference in the vascular density of the choriocapillaris. The vascular density of the choriocapillaris was decreased in more myopic eyes only when there was a variance of more than 1.50 D in the anisometropic participants. The repeatability of the choroidal thickness, vascular density, and flow voids measurements was comparable with other studies [25, 26]. The choriocapillaris was defined as the structure located from 9 μm above to 31 μm below Bruch’s membrane, consistent with Al-Sheikh et al.’s study [21], but different from Wu et al.’s. The latter analyzed the choriocapillaris 20 μm below the Bruch’s membrane. Therefore, the flow voids of the choriocapillaris results in this study are more than Wu et al.’s reports [25, 26].

Here, the choroidal thickness of the more myopic eyes was significantly thinner than less myopic eyes in the macular foveal and parafoveal regions. This is consistent with the results of previous studies [32, 33]. Vincent et al. [24] examined the choroidal thickness of more and less myopic eyes among a sample of myopic anisometropia. The authors found that in horizontal B-scan, the choroidal thickness was significantly thinner in the more myopic eyes than the less myopic eyes. The interocular difference in the SFCT was significantly correlated with the degree of axial anisometropia [24]. Further, several recent cross-sectional population-based studies have shown a negative correlation between the choroidal thickness and the severity of axial myopia [2, 24, 34–36]. Consistently, several longitudinal studies on children and adolescents have further confirmed choroidal thinning during myopia development [37–39]. Other studies have shown that the choroid plays an important role in the development of myopia, as evidenced by the bidirectional changes in choroidal thickness in response to imposed myopic or hyperopic defocusing, termed “choroidal accommodation” [5–8]. Therefore, choroidal thickness changes are associated with the development of myopia.

The choroid is a highly vascularized tissue consisting of three vascular layers (i.e., choriocapillaris and medium and large vessel layers). Choroid thinning mainly occurs in the medium and large vessel layers with myopia [40]. It is still unclear whether myopia-related choroidal thinning is accompanied by decreased choroidal blood flow. In the current study, the results revealed no significant difference in the vascular density of the choriocapillaris between the more and less myopic eyes of each patient with anisometropia. There was only a significant difference in the choroidal thickness and flow voids. Interestingly, the vascular density of the choriocapillaris in the more myopic eyes was significantly lower than less myopic eyes when anisometropia exceeded 1.50 D. This result indicates that the vascular density of choriocapillaris may not decrease in the early stage of myopia which is consistent with the results of Yazdani et al. [41], who observed a slightly higher choroidal vascularity index in low myopes compared with emmetropes.

Recently, several studies have reported that myopia-stimulating signals can reduce the perfusion of the choroid, leading to an insufficient supply of oxygen and nutrients to the nearby sclera. This would result in scleral hypoxia and thinning and ultimately cause axial elongation and myopia [12–14]. Does a decrease in choroidal circulation underly myopia development in anisometropia? Wu et al. demonstrated that the restructuring of the sclera in myopia was accompanied by large-scale transdifferentiating of scleral fibroblasts into myofibroblasts. Importantly, hypoxia-inducible factor-1α (HIF-1α) in the sclera plays a prominent role in signaling this restructuring, suggesting that a scleral hypoxia-dependent mechanism plays an important role in the underlying myopic development [12]. Zhang et al. found that choroidal thickness and choroidal blood perfusion (ChBP) were significantly decreased in guinea pig myopia. Further, changes in choroidal thickness were positively correlated with changes in ChBP [42]. Zhou et al. indicated that increased ChBP attenuated scleral hypoxia, thereby inhibiting the development of myopia. Therefore, ChBP may serve as an immediate predictor of myopia development [43].

The results of this study indicated that the vascular density of the choriocapillaris was decreased in the more myopic eyes of anisometropia when a cutoff value of 1.50 D (with a mean interocular difference in SER − 2.34 ± 0.62 D) was adopted compared to the less myopic eyes of anisometropia. Several recent studies have found that the area of the choriocapillaris without perfusion is increased in highly myopic eyes compared to controls with low myopia or emmetropia. This indicates that differences in choriocapillaris blood flow are more apparent between groups with larger differences in myopia [20, 21]. In addition, Shih et al. used the ocular pulse amplitude (OPA) and pulsatile ocular blood flow (POBF) to indirectly measure choroidal blood flow. In anisometropic participants with a variance of more than 2.00 D, the OPA and POBF were significantly reduced in the more myopic eyes [44–48], further indicating the presence of decreased choriocapillaris blood flow in the more myopic eyes of myopic anisometropic patients. These patients are likely to exhibit the greatest amount of axial elongation. Gupta et al. [49] reported that choroidal thinning in highly myopic eyes was accompanied by reductions in the contents of vascular tissue and stromal tissue. Collectively, these findings suggest that the choroidal vascular volume is more likely to decrease with myopia worsening, suggesting worsened choroidal circulation in myopic eyes.

This study has several limitations. First, we adopted a cross-sectional design; future longitudinal studies should clarify the association between reduced choroidal thickness and myopia development. Another limitation is that the design did not include enough monocular myopic and binocular myopic anisometropic participants; the homogeneous sample may be the reason why some differences were not statistically significant. Finally, there were technical limitations. For example, using SD-OCT failed to simultaneously analyze the vascular structure quickly and clearly. Further studies using high-speed SS-OCT system should improve choroidal vasculature imaging and choroidal angiography.

## Conclusions

In conclusion, the choroidal thickness on both horizontal and vertical B-scans was thinner in the more myopic eyes and was negatively correlated with the degree of axial myopia. The flow voids increased, and the vascular density of the choriocapillaris was decreased in the more myopic eyes of patients with anisometropia exceeding 1.50 D. The association between myopia and choroidal blood flow requires further investigation.

## Data Availability

The datasets used and/or analysed during the current study are available from the corresponding author on reasonable request.

## References

[1] Holden BA, Fricke TR, Wilson DA, Jong M, Naidoo KS, Sankaridurg P (2016). Global prevalence of myopia and high myopia and temporal trends from 2000 through 2050. Ophthalmology.

[2] Jin PY, Zou HD, Zhu JF, Xu X, Jin J, Chang TC (2016). Choroidal and retinal thickness in children with different refractive status measured by swept-source optical coherence tomography. Am J Ophthalmol.

[3] Flores-Moreno I, Lugo F, Duker JS, Ruiz-Moreno JM (2013). The relationship between axial length and choroidal thickness in eyes with high myopia. Am J Ophthalmol.

[4] Nickla DL, Wallman J (2010). The multifunctional choroid. Prog Retin Eye Res.

[5] Chiang ST, Phillips JR, Backhouse S (2015). Effect of retinal image defocus on the thickness of the human choroid. Ophthalmic Physiol Opt.

[6] Zhu XY, Park TW, Winawer J, Wallman J (2005). In a matter of minutes, the eye can know which way to grow. Invest Ophthalmol Vis Sci.

[7] Rada JA, Nickla DL, Troilo D (2000). Decreased proteoglycan synthesis associated with form deprivation myopia in mature primate eyes. Invest Ophthalmol Vis Sci.

[8] Wildsoet C, Wallman J (1995). Choroidal and scleral mechanisms of compensation for spectacle lenses in chicks. Vis Res.

[9] Woodman-Pieterse EC, Read SA, Collins MJ, Alonso-Caneiro D (2015). Regional changes in choroidal thickness associated with accommodation. Invest Ophthalmol Vis Sci.

[10] Chen Z, Xue F, Zhou J, Qu X, Zhou X (2016). Effects of orthokeratology on choroidal thickness and axial length. Optom Vis Sci.

[11] Lee JH, Hong IH, Lee TY, Han JR, Jeon GS (2021). Choroidal thickness changes after orthokeratology lens wearing in young adults with myopia. Ophthalmic Res.

[12] Wu H, Chen W, Zhao F, Zhou Q, Reinach PS, Deng L (2018). Scleral hypoxia is a target for myopia control. Proc Natl Acad Sci USA.

[13] Fitzgerald ME, Wildsoet CF, Reiner A (2002). Temporal relationship of choroidal blood flow and thickness changes during recovery from form deprivation myopia in chicks. Exp Eye Res.

[14] Wu PC, Tsai CL, Gordon GM, Jeong S, Itakura T, Patel N (2015). Chondrogenesis in scleral stem/progenitor cells and its association with form-deprived myopia in mice. Mol Vis.

[15] Wang Q, Chan S, Yang JY, You B, Wang YX, Jonas JB (2016). Vascular density in retina and choriocapillaris as measured by optical coherence tomography angiography. Am J Ophthalmol.

[16] Margolis R, Spaide RF (2009). A pilot study of enhanced depth imaging optical coherence tomography of the choroid in normal eyes. Am J Ophthalmol.

[17] Sohrab M, Wu K, Fawzi AA (2012). A pilot study of morphometric analysis of choroidal vasculature in vivo, using en face optical coherence tomography. PLoS One.

[18] Chirco KR, Sohn EH, Stone EM, Tucker BA, Mullins RF (2017). Structural and molecular changes in the aging choroid: implications for age-related macular degeneration. Eye (Lond).

[19] Cicinelli MV, Rabiolo A, Marchese A, de Vitis L, Carnevali A, Querques L (2017). Choroid morphometric analysis in non-neovascular age-related macular degeneration by means of optical coherence tomography angiography. Br J Ophthalmol.

[20] Su L, Ji Y, Tong N, Sarraf D, He X, Sun X (2020). Quantitative assessment of the retinal microvasculature and choriocapillaris in myopic patients using swept-source optical coherence tomography angiography. Graefes Arch Clin Exp Ophthalmol.

[21] Al-Sheikh M, Phasukkijwatana N, Dolz-Marco R, Rahimi M, Iafe NA, Freund KB (2017). Quantitative OCT angiography of the retinal microvasculature and the choriocapillaris in myopic eyes. Invest Ophthalmol Vis Sci.

[22] Milani P, Montesano G, Rossetti L, Bergamini F, Pece A (2018). Vessel density, retinal thickness, and choriocapillaris vascular flow in myopic eyes on OCT angiography. Graefes Arch Clin Exp Ophthalmol.

[23] Vincent SJ, Collins MJ, Read SA, Carney LG (2014). Myopic anisometropia: ocular characteristics and aetiological considerations. Clin Exp Optom.

[24] Vincent SJ, Collins MJ, Read SA, Carney LG (2013). Retinal and choroidal thickness in myopic anisometropia. Invest Ophthalmol Vis Sci.

[25] Wu H, Xie Z, Wang P, Liu M, Wang Y, Zhu J (2021). Differences in retinal and choroidal vasculature and perfusion related to axial length in pediatric anisomyopes. Invest Ophthalmol Vis Sci.

[26] Wu H, Zhang G, Shen M, Xu R, Wang P, Guan Z (2021). Assessment of choroidal vascularity and choriocapillaris blood perfusion in anisomyopic adults by SS-OCT/OCTA. Invest Ophthalmol Vis Sci.

[27] Hoseini-Yazdi H, Vincent SJ, Read SA, Collins MJ (2020). Astigmatic defocus leads to short-term changes in human choroidal thickness. Invest Ophthalmol Vis Sci.

[28] Tan CS, Ouyang Y, Ruiz H, Sadda SR (2012). Diurnal variation of choroidal thickness in normal, healthy subjects measured by spectral domain optical coherence tomography. Invest Ophthalmol Vis Sci.

[29] Bennett AG, Rudnicka AR, Edgar DF (1994). Improvements on Littmann's method of determining the size of retinal features by fundus photography. Graefes Arch Clin Exp Ophthalmol.

[30] Tian J, Marziliano P, Baskaran M, Tun TA, Aung T (2013). Automatic segmentation of the choroid in enhanced depth imaging optical coherence tomography images. Biomed Opt Express.

[31] Zhang Q, Zheng F, Motulsky EH, Gregori G, Chu Z, Chen CL (2018). A novel strategy for quantifying choriocapillaris flow voids using swept-source OCT angiography. Invest Ophthalmol Vis Sci.

[32] El-Shazly AA, Farweez YA, ElSebaay ME, El-Zawahry WMA (2017). Correlation between choroidal thickness and degree of myopia assessed with enhanced depth imaging optical coherence tomography. Eur J Ophthalmol.

[33] Li T, Zhou X, Wang Z, Zhu J, Shen W, Jiang B (2016). Assessment of retinal and choroidal measurements in Chinese school-age children with Cirrus-HD optical coherence tomography. PLoS One.

[34] Wei WB, Xu L, Jonas JB, Shao L, Du KF, Wang S (2013). Subfoveal choroidal thickness: the Beijing Eye Study. Ophthalmology.

[35] Read SA, Collins MJ, Vincent SJ, Alonso-Caneiro D (2013). Choroidal thickness in myopic and nonmyopic children assessed with enhanced depth imaging optical coherence tomography. Invest Ophthalmol Vis Sci.

[36] Tan CS, Cheong KX (2014). Macular choroidal thicknesses in healthy adults–relationship with ocular and demographic factors. Invest Ophthalmol Vis Sci.

[37] Read SA, Alonso-Caneiro D, Vincent SJ, Collins MJ (2015). Longitudinal changes in choroidal thickness and eye growth in childhood. Invest Ophthalmol Vis Sci.

[38] Jin P, Zou H, Xu X, Chang TC, Zhu J, Deng J (2019). Longitudinal changes in choroidal and retinal thicknesses in children with myopic shift. Retina.

[39] Xiong S, He X, Zhang B, Deng J, Wang J, Lv M (2020). Changes in choroidal thickness varied by age and refraction in children and adolescents: a 1-year longitudinal study. Am J Ophthalmol.

[40] Devarajan K, Sim R, Chua J, Wong CW, Matsumura S, Htoon HM (2020). Optical coherence tomography angiography for the assessment of choroidal vasculature in high myopia. Br J Ophthalmol.

[41] Yazdani N, Ehsaei A, Hoseini-Yazdi H, Shoeibi N, Alonso-Caneiro D, Collins MJ (2021). Wide-field choroidal thickness and vascularity index in myopes and emmetropes. Ophthalmic Physiol Opt.

[42] Zhang S, Zhang G, Zhou X, Xu R, Wang S, Guan Z (2019). Changes in choroidal thickness and choroidal blood perfusion in guinea pig myopia. Invest Ophthalmol Vis Sci.

[43] Zhou X, Zhang S, Zhang G, Chen Y, Lei Y, Xiang J (2020). Increased choroidal blood perfusion can inhibit form deprivation myopia in guinea pigs. Invest Ophthalmol Vis Sci.

[44] Shih YF, Horng IH, Yang CH, Lin LL, Peng Y, Hung PT (1991). Ocular pulse amplitude in myopia. J Ocul Pharmacol.

[45] Lam AK, Chan ST, Chan B, Chan H (2003). The effect of axial length on ocular blood flow assessment in anisometropes. Ophthalmic Physiol Opt.

[46] Kaufmann C, Bachmann LM, Robert YC, Thiel MA (2006). Ocular pulse amplitude in healthy subjects as measured by dynamic contour tonometry. Arch Ophthalmol.

[47] Baxter GM, Williamson TH (1995). Color Doppler imaging of the eye: normal ranges, reproducibility, and observer variation. J Ultrasound Med.

[48] Quaranta L, Harris A, Donato F, Cassamali M, Semeraro F, Nascimbeni G (1997). Color Doppler imaging of ophthalmic artery blood flow velocity: a study of repeatability and agreement. Ophthalmology.

[49] Gupta P, Thakku G, Saw SM, Tan M, Lim E, Tan M (2017). Characterization of choroidal morphologic and vascular features in young men with high myopia using spectral-domain optical coherence tomography. Am J Ophthalmol.

